# Raman Spectroscopy in the Characterization of Food Carotenoids: Challenges and Prospects

**DOI:** 10.3390/foods14060953

**Published:** 2025-03-11

**Authors:** Stefan M. Kolašinac, Ilinka Pećinar, Radoš Gajić, Dragosav Mutavdžić, Zora P. Dajić Stevanović

**Affiliations:** 1 Department of Agrobotany, Faculty of Agriculture, University of Belgrade, Nemanjina 6, Zemun, 11080 Belgrade, Serbia; ilinka@agrif.bg.ac.rs (I.P.); dajic@agrif.bg.ac.rs (Z.P.D.S.); 2 Institute of Physics, Centre for Solid State Physics and New Materials, P.O. Box 68, Pregrevica 118, 11080 Belgrade, Serbia; rgajic@ipb.ac.rs; 3 Institute for Multidisciplinary Research, University of Belgrade, Kneza Višeslava 1, 11030 Belgrade, Serbia; gane@imsi.bg.ac.rs

**Keywords:** Raman spectroscopy, AI-assisted spectroscopy, carotenoids, food

## Abstract

This paper presents an overview of the application of Raman spectroscopy (RS) in characterizing carotenoids, which have recently gained attention due to new findings on their health-promoting effects and rising demand in the food, pharmaceutical, and cosmetic industries. The backbone structure in the form of a polyene chain makes carotenoids sensitive to Raman spectroscopy, mainly due to the stretching vibrations of their conjugated double bonds. Raman spectroscopy is increasingly used in agricultural and food sciences and technologies as it is a non-preparative, environmentally friendly, fast and efficient method for characterizing target analytes. The application of RS in the qualitative and quantitative analysis of carotenoids requires the careful selection and adjustment of various instrument parameters (e.g., laser wavelength, laser power, spectral resolution, detector type, etc.) as well as performing complex chemometric modeling to interpret the Raman spectra. Most of the studies covered in this review focus more on qualitative than quantitative analysis. The most frequently used laser wavelengths are 1064, 785, and 532 nm, while 633 nm is the least used. Considering the sensitivity and complexity of RS, the present study focuses on the specific and critical points in the analysis of carotenoids by RS. The main methodological and experimental principles in the study of food carotenoids by RS are discussed and best practices recommended, while the future prospects and expectations for a wider application of RS, especially in food quality assessment, are emphasized. New Raman techniques such as Spatially Offset Raman Spectroscopy (SORS), Coherent Anti-Stokes Raman Spectroscopy (CARS) and Stimulated Raman Scattering Spectroscopy (SRS), as well as the application of artificial intelligence, are also described in the context of carotenoids analysis.

## 1. Microscopic Theory

**Figure 1:**
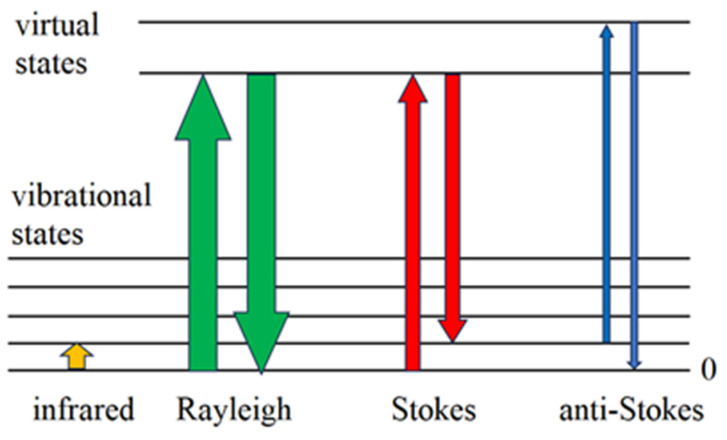
Energy levels and Raman intensities of the states involved in Raman scattering. The levels of intensities are symbolically represented by arrow thickness.

Light scattering could be elastic (Rayleigh scattering), when the incoming and outgoing radiation are of the same frequency, or inelastic (Raman scattering), where the frequency of outgoing light is shifted due to the creation or absorption of excitations such as phonons (see Figure 1) [1]. In contrast to IR spectroscopy, in Raman spectroscopy, phonons are indirectly excited. Since Raman scattering is very weak, an intense light source, a laser, is required. The Raman intensities are 10^−6^ to 10^−8^ of laser light. Actually, the Raman spectroscopy of solids became possible only after invention of lasers. In most cases, Raman scattering measurements are carried out with visible lasers. The small probabilities of Raman scattering can be properly explained using a quantum mechanical description of the Raman scattering process.

**Figure 2:**
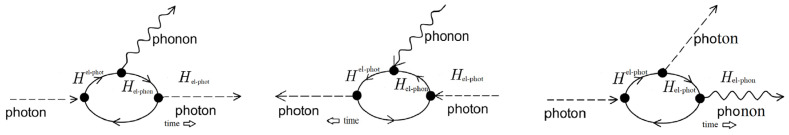
Feynman diagram of the Stokes (**left**), its time-reversed—anti-Stokes—Raman (**middle**) and an additional indistinguishable Stokes Feynman diagram (**right**).

Raman scattering is rather intricate, depending upon the third-order time-dependent perturbation theory, including all possible intermediate (virtual) states. A typical three-vertex Feynman diagram is shown in Figure 2.

**Figure 3:**
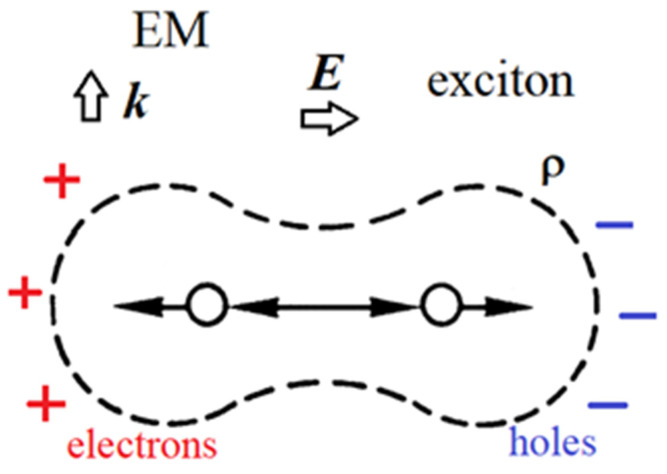
The vibration of a diatomic molecule in the laser field gives rise to the electronic distribution, whereas the arrows within the molecule are vibrational displacements.

For most people who perform Raman measurements, virtual states are usually an unclear notion. These are not stationary states of the system nor a solution of the time-independent Schrodinger equation, and therefore do not correspond to well-defined energy. Short-lived, unobservable, timeless states are the mathematical construct used for calculating transitions between initial and final states [2]. As an example, let us consider a diatomic molecule under laser radiation directed upwards (the k vector) (Figure 3) [3]. The electric field E of the incoming laser light (ω__L_ in the initial state) has an energy much higher than the vibrational energies, and interacts just with the electrons. It separates the charges, producing extra electrons and holes at the ends of the molecule (exciton), as depicted in Figure 3.

As an example, let us analyze the Stokes Raman Feynman diagram in Figure 2.

- First vertex: The incident photon interacts with an electron in the molecule creating an intermediate (virtual) state of an electron–hole pair (or exciton).
- Second vertex 2: This electron–hole pair is scattered into another virtual state by emitting a phonon via the electron–phonon interaction.
- Third vertex 3: Due to the electron–phonon interaction, the electron–hole pair recombines with the emission of the scattered photon.

Actually, each possible Feynman diagram described above represents the wave function (or the probability amplitude) for an alternative indistinguishable mode of Raman scattering. There are six alternative ways (diagrams), and the overall wave function of the Raman scattering will be the sum of each indistinguishable way considered separately. The probability (proportional to the intensity) will be the square of this sum. Practically speaking, this is the only thing one needs to know about quantum mechanics: if it is not possible to distinguish different internal ways, then all separate wave functions must be summed, and the square of the sum is the probability of a process being observed. Otherwise, the separate probabilities must be summed for each distinguished way [4].

Thus, electrons mediating the Raman scattering of phonons remain unchanged after the process. Since the transitions involving the electrons are virtual, they do not have to conserve energy, although they still have to conserve wavevectors.

**Figure 4:**
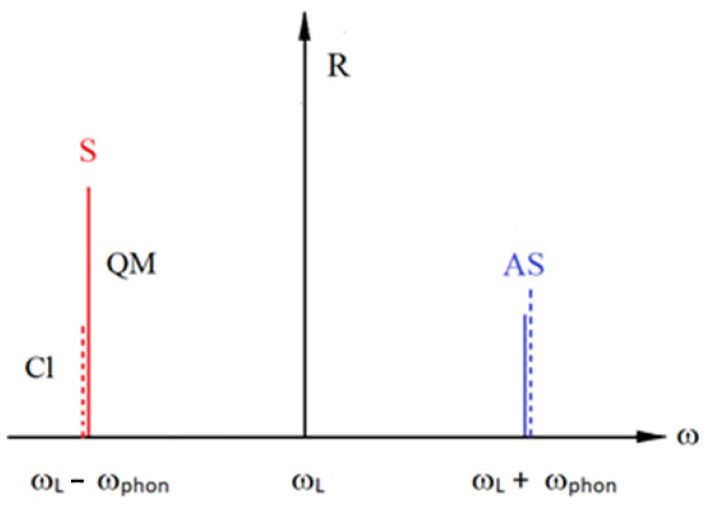
Stokes and anti-Stokes intensities. The full and dashed lines correspond to quantum and classical theory, respectively.

In the Stokes Raman scattering process shown in Figure 2, one phonon is created when the scattered laser light has the frequency ω__L_ − ω_phon_ (Figure 1). Stokes Raman scattering does not require any phonon be present in the initial state, as is the case for T = 0 K. For temperatures different from zero, there is a probability of an already-excited phonon being present in the initial state, and additionally, anti-Stokes Raman scattering occurs when the excited phonon is annihilated and the scattered laser light has ω__L_ + ω_phon_ (Figure 1). The explanation for this follows the same reasoning as before. Actually, the anti-Stokes Raman process is just time-reversed Stokes Raman scattering, as shown in Figure 2. In both cases, it is necessary to sum the contributions of the six different indistinguishable processes that are described by similar Feynman diagrams, as in Figure 2. The squared sum of all six processes is proportional to the scattering probability, i.e., the intensity of Raman scattering. In practice, it is quite difficult to calculate the Raman intensities precisely, since they depend on material parameters, as well as structural and laser characteristics. However, it is possible to calculate the ratio of Stokes and anti-Stokes Raman intensities precisely. The classical treatment gives (I_Stokes_/I_anti-Stokes_)_class_ = (ω__L_ − ω_phon_)^4^/( ω__L_ + ω_phon_)^4^—denoted by the dashed line in Figure 4—and is not in agreement with the experimental results at all. On the other hand, a simple classical explanation is successful in predicting the existence of Stokes and anti-Stokes Raman scattering. Regarding the Raman intensities, a proper quantum mechanical treatment is necessary. The probabilities of the Stokes and anti-Stokes scatterings explained before are the same probabilities as seen for the creation or annihilation of just one phonon. If more phonons are involved, I_Stokes_ should be multiplied by the factor n + 1, and I_anti-Stokes_ by n. n is the number of phonons, and is given as n = 1/exp(hν/k_B_T— − 1), as phonons are bosons and satisfy the Bose–Einstein distribution. Here, h is the Planck constant, ν the frequency of the phonon and k_B_ the Bolzman constant. Therefore, the ratio of the intensities I_Stokes_/I_anti-Stokes_ = (I_Stokes_/I_anti-Stokes_)_class_ · (n + 1)/n = (I_Stokes_/I_anti-Stokes_)_class_ · exp(hν/k_B_T) (Figure 4). It is worth mentioning that the anti-Stokes lines do not exist at T = 0, since there are no phonons at T = 0. Also, the previous formula enables the determination of the sample temperature, provided that we know the intensities of both the Stokes and anti-Stokes Raman lines (the Raman thermometry).

The existence of Stokes and anti-Stokes Raman scattering also has a simple classical explanation [3], whereas the Raman intensities require a proper quantum mechanical treatment, as explained by the fact that the classical explanation is not in agreement with the experimental findingns (Figure 4).

## 2. Overview and Importance of Plant Carotenoids

Bioactive molecules as important components of fruits, vegetables and whole grains have been extensively studied for their beneficial prophylactic and curative effects. Among plant secondary metabolites, carotenoids are known as an evolutionarily old group of lipophilic pigments playing various roles in plant metabolism, development and stress responses (e.g., [5,6]). Carotenoids are synthesized in photosynthetic organisms such as algae, cyanobacteria and plants, but have also been found in some non-photosynthetic organisms, including some bacteria, fungi, and yeasts [7,8]. In plants and other photosynthetic organisms, carotenoids are essential for photosynthesis and photoprotection [9]. These pigments are able to increase light absorption in the blue spectral range of 450–570 nm, transferring the energy to chlorophyll, and channeling the excess energy of chlorophyll to protect the photosynthetic apparatus from reactive oxygen species (ROS) [7,8]. The oxidative cleavage of carotenoids results in the appearance of apocarotenoids known as crucial plant signaling molecules, such as the hormone abscisic acid (ABA) [10]. In contrast to the long chain carotenoids (C40 and >40), apocarotenoids are shorter molecules derived by the modification of a parental molecule by carotenoid cleavage dioxigenases (CCDs) [11]. Carotenoids, together with chlorophylls, are associated with proteins, and serve as accessory pigments embedded in the thylakoid membranes of chloroplasts, but they are also frequently found in chromoplasts in the forms of crystalline, globular, tubular and fibrilar shapes, usually mixed with cell lipids [12,13]. The functions of carotenoids include the attraction of animals for pollination, the dispersal of fruits and seeds thanks to the intense color of the petals, and the ripening of fruits due to pericarp-accumulated carotenoids [13].

There are more than 1000 carotenoids with natural structural variants, of which about 40 have been identified, in a typical human diet [14]. In vascular plants, there are two types of carotenoids; non-oxygenated carotenoids, called carotenes (such as β-carotene and lycopene), and the oxygenated derivatives of carotenoids, such as astaxanthin, lutein, violaxanthin, neoxanthin, and zeaxanthin, known as xanthophylls or phylloxanthins, depending on the absence or presence of oxygen groups in their molecular structure [12,15].

Both carotenes and xanthophylls are present in the human diet mainly in form of the C40-based isoprenoids, originating from various fruit, vegetables, cereals, spices and edible flowers. Xanthophylls (primarily lutein and zeaxanthin) are usually found in leafy vegetables, as well as in peas, durum wheat, and maize [16,17]. The β-Cryptoxanthin is found in citrus fruits, melons, guavas, apples, and pumpkins, imparting yellow and orange colors, whereas capsanthin and capsorubin are typical red pigments of peppers [18,19]. Carotenes are responsible for the red color of tomatoes, watermelons, and papaya (due to lycopene), and for the orange color of carrots, oranges, pumpkin, sweet potatoes, and mangos (due to α- and β-carotene) [20,21]. Violaxanthin (yellow and orange) is abundant in the petals of many flowers (e.g., lily, gerbera, calendula), as well as in mango fruit and potato tubers [22]. Neoxanthin and crocin, providing a yellow color, are found in some leafy vegetables and saffron stigma, respectively [12,14]. Due to their traits and high biological activity, carotenoids are widely applied in food coloring, fortified and functional foods, animal feed, dietary supplements, pharmaceuticals, and cosmetics. According to the BCC Publishing Research Team report for 2022, the global carotenoids market should increase from USD 2.0 billion to USD 2.7 billion by 2027, with an annual growth rate (CAGR) of 5.7%, driven by the demand for natural colorants, additives, and novel pharmaceutical and personal care products. Carotenoids are acknowledged for their health-related benefits, such as their antioxidant, antimicrobial and anticancer activity, immune system-strengthening, skin protection, neurological and cognitive disorder (including Alzheimer) amelioration, osteoporosis, and anti-diabetic, anti-obesity and anti-age effects (e.g., [8,23]). The beneficial effects of carotenoid-rich plant species in reducing the risks of certain diseases are attributed to major carotenoids such as β-carotene, lycopene, lutein, zeaxanthin, and crocetin due to their high antioxidant activity.

**Figure 5:**
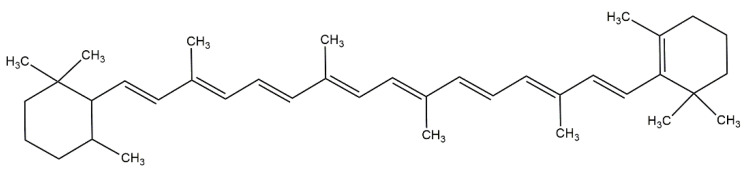
Polyene backbone structure with conjugated double bonds system (β-carotene).

The health-beneficial effects of carotenoids are a consequence of their polyene backbone structure with a conjugated double-bonds system (Figure 5), which stabilizes the unpaired electrons during the ROS scavenging processes [24,25]. The biosynthesis of carotenoids is performed via the plastid methylerythritol (MEP) pathway, starting with the condensation of two geranylgeranyl pyrophosphate (GGPP) molecules (initially formed from the binding of isopentenyl diphosphate—IPP, C5—with its isomere, the dimethylallyl diphosphate DMAPP, C5), which results in the occurrence of the first colorless phytoene [26].

Carotenoids are hydrophobic (low-water-soluble) molecules sensitive to oxidation under exposure to oxygen, light, heat, pH alterations, and the presence of acids and metal ions [27]. Due to their chemical instability and frequent cis-trans isomerization, the use of standard analytical techniques in the precise identification and quantification of carotenoids is still challenging.

## 3. In Situ Raman Spectroscopy Determination and Quantification of Foods Carotenoids

The literature has described different analytical techniques used for the qualitative and quantitative identification of carotenoids. The gold standard analytical methods for measuring carotenoid concentration are high-performance liquid chromatography (HPLC) [19], high-performance thin-layer chromatography (HPTLC) [28] and NMR spectroscopy [29], in addition to some advanced analytical tools such as liquid chromatography–tandem mass spectrometry (LC-MS/MS), ultra-high-performance liquid chromatography (UHPLC), and matrix-assisted laser desorption ionization time-of-flight mass spectrometry (MALDI/TOF-MS) [19,30]. These analytical methods are time-consuming, requiring lengthy standardization procedures, the selection of the best solvents and extraction procedures, and the solving of the problem of their low stability, as well as the difficultly of isolating and chemically transforming carotenoids during extraction processes [31]. To mitigate these issues, carotenoid-rich samples (such as fresh fruit, lyophilized samples, or extracts) should be stored at −80 °C to preserve their stability. The extraction procedure should be performed quickly, at room temperature and in the dark, to prevent the degradation, oxidation, or isomerization of carotenoids caused by heat, light, or oxygen exposure [32].

To overcome all the limitations on the performance of standard chemical analytical techniques, vibrational spectroscopy methods have been developed, allowing the research of various types of samples, including plant metabolites, under in situ and in vivo conditions [33]. Raman spectroscopy (RS) is a non-destructive, time-saving and environmentally friendly analytical tool preferred for the monitoring, imaging, and characterization of non-biological and biological samples and their components. Unlike conventional analytical methods, RS is a technique for directly measuring specific vibrational motions of molecules, and it can be used to determine the molecular structures of a wide range of compounds, providing important information about the nature of chemical bonds and the intermolecular forces acting between the atoms of a molecule [33]. Raman spectroscopy works on the principle of an inelastic light-scattering phenomenon known as Raman scattering (Raman effect) for the identification of analytes by their molecular bond vibrations (e.g., [34]).

Further, Raman spectroscopy has considerable advantages that are important for food analysis, e.g., high specificity and the yielding of unique fingerprint structure information, making it a more practical analytical tool. Raman spectroscopy can be used to complete the analysis in a few seconds, and is thus able to monitor industrial processes in real time and analyze water-rich samples such as food, given that the peak intensities of water molecules are outside the fingerprint range [35].

Raman imaging can provide deeper insights into mechanisms during food analysis. Compared to various standard analytical methods and imaging techniques, Raman imaging technology provides more detailed information about the vibrations of covalent bonds and enables the better visualization of target substances in food, allowing more accurate observation of their presence and spatial distribution. Moreover, it can be used at the micro- or nanoscale, overcoming the limitations of hyperspectral imaging methods, which are usually restricted to macroscopic sample evaluations [36].

The results of the application of RS are encouraging, and we anticipate that RS can be used to replace conventional methods, and play an important role in the quality and safety control of agricultural products and food in the future. Finally, the combination of RS with artificial intelligence technology will expand the application of the Raman technique, which is of great significance for building a food safety monitoring network covering agriculture, the food industry, and the freshness of agricultural products after harvest and in the market [37].

**Figure 6:**
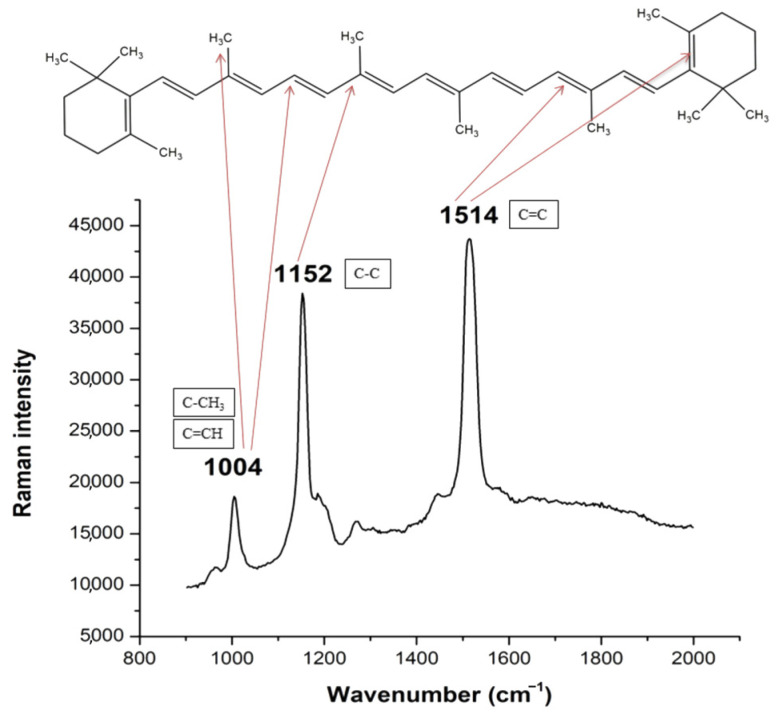
Raman spectrum of carotenoids. Raman profiles show common bands at 1004 cm^−1^ and 1152 cm^−1^ associated with C-CH_3_ in-plane rocking and C–C stretching, respectively. The band at 1514 cm^−1^ corresponds to C=C stretching vibration (performed on Horiba XploRA, 532 nm laser).

Raman spectroscopy is a particularly valuable tool for the identification, localization and quantification of molecules containing at least one double bond. Due to the presence of conjugated carbon single and double bonds causing high levels of stretching vibrational motion, the carotenoids are among the most visible nutrients in the fingerprint region of the Raman spectrum (Figure 6). The conjugated double bond system in carotenoids significantly enhances their Raman signal intensity through resonance effects, making Raman spectroscopy an excellent tool for the qualitative and quantitative analysis of carotenoids in food, pharmaceuticals, and biological samples [38].

The micro-Raman single-point and related mapping technique could be used to provide detailed information about the relative contents, distributions, and in situ tissue-specific accumulation of carotenoids in different samples (Table 1 and Table 2). The identification of carotenoids in biological systems by Raman spectroscopy is achieved by analyzing the three main characteristic Raman bands. The positions of the carotenoids’ bands are specific, and depend on the structure of carotenoid terminal and functional (in the case of xanthophylls) groups, the number of covalent (especially double) bonds, as well as interactions with other plant components [14]. The Raman spectra of carotenoids are characterized by slight structural differences [39]. The Raman spectra of different carotenoid-rich samples show two main bands arising in the 1500–1570 cm^−1^ range due to the ν(C=C) vibrational mode, and in the 1130–1172 cm^−1^ range due to the ν(C-C) mode of the conjugated polyene chain [39,40]. The wavenumbers of the C=C and C-C stretching vibrations reflect the degree of conjugation, with the wavenumber gradually increasing as the length of the conjugated bond decreases [21]. The band in the range of 1005–1020 cm^−1^ originates from the in-plane rocking motions of the -CH_3_ group bound to the polyene chain showing moderate intensity [41].

Raman spectroscopy has several limitations when applied to carotenoid analysis, particularly due to the complexity of food matrices, which often contain a variety of primary and secondary metabolites. While Raman spectroscopy is sensitive to carotenoids, detecting specific carotenoids, such as β-carotene, α-carotene, or lycopene, in complex matrices like tomato fruit remains challenging. The complexity of these samples results in intricate and overlapping spectra, which necessitate the use of chemometric methods to extract meaningful information. The ability to transform spectra, as well as the application of linear and non-linear regression and classification models, is crucial for effective analysis. Additionally, the sensitivity of Raman instrumentation can lead to variability in the results from day to day. To achieve stable and repeatable models, advanced statistical knowledge is required to account for and correct within-group variations [42,43].

Carotenoids have been studied since the 1970s [44,45]. The application of RS in analyzing carotenoids in food samples, such as tomato fruits and products, paprika, and carrot, was first reported by Baranska et al. (2006) [46], Baranski et al. (2005) [47] and Schulz et al. (2005) [48]. Since then, investigations have covered a lot of carotenoids-rich plant species and/or their products, both those with vivid colors, such as corn, sweet potato, apricot, etc., as well as green leafy vegetables, in which the carotenoids are masked by chlorophyll pigments (see Table 1 and Table 2). In most cases, the single point measurement method was applied, aiming to confirm the presence of the expected carotene and/or xanthophyll molecule by assignment the characteristic Raman bands [40,49]. Raman imaging (2D or 3D) is usually performed in studies on carotenoids’ occurrence, accumulation, and distribution along a spatial gradient of the studied material, i.e., tissue [50], mainly to comprehend the locations of carotenoids’ biosynthesis, translocation and localization [51]. The most recent approaches applied in studies of carotenoids involved the use of Surface Enhanced Raman Spectroscopy (SERS). SERS is an emerging Raman technique with an expected high applicability in more sophisticated analyses of carotenoids by RS [52]. Generally, SERS accounts for electromagnetic and chemical enhancements provided by different substrates, of which gold and silver have mostly been used (e.g., [53]). Zhou and Kneipp (2023) [54] showed that SERS performed with silver nanoparticles could enable the differentiation of the two carotenoid molecules—β-carotene and trans-β-Apo-8′-carotenal.

The application of RS-coupled chemometrics seems promising. For example, RS saw practical application in carotenoid measurements in food samples by successfully discriminating different groups (classes). For example, Akpolat et al. (2020) [55] used the discrimination model Soft Independent Modelling of Class Analogy (SIMCA) to separate different tomato genotypes, while Wang et al. (2021) [21] conducted a similar investigation through measurements of the impact of exposure time on discrimination power. Kolašinac et al. (2021) [56] conducted a study on using RS in the discrimination/identification of successive ripening phases during red pepper fruit development. The results show that RS in combination with appropriate chemometric modeling approaches, such as SIMCA, can efficiently discriminate samples with absolute accuracy (precision of 100%). Farber et al. (2020) [57] used RS to discriminate non-infected and infected wheat samples. RS in combination with a selected multivariate statistical model (Orthogonal Partial Least Squares Discriminant Analysis—OPLS-DA) showed potential use in the efficient discrimination of different peanut genotypes [57] (Table 1).

Hara et al. (2021) [20] investigated the potential use of RS equipped with a 785 nm laser to perform a quantitative analysis of the total carotenoids in tomato samples. RS was also used in the assessment of the extra virgin oil (EVOO) quality by measuring the lutein/β-carotene ratio [58]. The quantification of total carotenoids using RS was performed on sweet potato [59], falso guarana [60] and carrot samples [61]. Baranska et al. (2006) [46] performed quantitative analyses of the lycopene and β-carotene in tomato samples, showing high regression coefficient values (R^2^ = 0.91 and R^2^ = 0.89 for lycopene and β-carotene, respectively) (see Table 1 and Table 2 for details).

## 4. Statistical Processing of Raman Spectra for Qualitative and Quantitative Characterization of Carotenoids

Processed and raw food materials are in fact complex chemical matrixes (mixtures) consisting of a range of different molecules. Hence, the identification of a single component in the matrix by RS is difficult, and interpretation is challenging. Modern technological development and software diversity provide an opportunity to deal with large datasets, as well as tools to process them and extract the useful information. When performing statistical analyses aiming to interpret Raman spectra, several important issues should be taken into account to ensure accurate and meaningful results. The first concerns visualizing the spectra—the visualization of spectra gives preliminary information about the presence and position of expected bands of carotenoids, the presence of spikes, the amount of noise, etc. Generally, all statistical methods used in Raman spectra interpretation can be categorized as (a) pre-processing, (b) modeling, or (c) validation. The main principles of the statistical (chemometric) analysis of the Raman spectra of carotenoids are the same and valid for any other analyte [42,43].

The complexity of a statistical analysis depends on the aim of the investigation. If the aim is the simple confirmation of the presence of carotenoids in a sample, or a comparison of different carotenoid-rich materials, pre-processing analysis is sufficient. However, if one is attempting to quantify particular carotenoids, especially in complex food systems, there is a need to select, test and validate the best-fitting quantification models.

Pre-processing is a procedure that should improve the quality of spectra by eliminating signals such as fluorescence, cosmic rays, laser power fluctuation, detector noise, signals from the vessel in which the sample is located, etc. [62]. The most common pre-processing procedures are as follows:

- Baseline correction is a pre-processing algorithm used to separate true spectroscopic signals from background effects [63];
- Normalization is a very important part of a pre-processing procedure, especially when samples are measured under different experimental conditions, allowing unbiased analysis [64];
- Smoothing is one of the appropriate mathematical techniques used to reduce the noise in the spectra while preserving all the important spectral information of the analyte. There are a number of methods that can be used to smooth the spectra, even if this involves a distortion of some spectral features [65];
- Spectral calibration is a method that can be used to correct for the occurrence of wavelength shifts. Accurate calibration is essential for comparing and combining spectra obtained from different measurements;
- Outlier detection is the procedure of identifying and handling outliers in the spectra that could significantly impact the validity of further statistical analyses. Outliers may arise from instrument noise, sample artefacts, or from measurement errors;
- Feature selection helps identify relevant spectral characteristics for the analysis, considering the specific information needed for the study. The selection of appropriate variables is required to avoid over-fitting, as well as for improvements in the model’s interpretability.

Modeling involves regression and classification algorithms. Regression models are applied to quantify the contents of carotenoids in a sample; the most widely used are simple regression methods and multivariate regression models, such as Partial Least Square Regression (PLSR), Support Vector Machine Regression (SVMR), Multivariate Linear Regression (MLR), etc. (Table 2). Classification models serve to group spectra via similarities in their chemical structure, i.e., corresponding with the Raman bands of the studied analyte; such models are divided into supervised (e.g., PLS, SVM, PLS-DA, etc.) and unsupervised (e.g., PCA).

Cross-validation involves the implementation of the cross-validation techniques required for generalized statistical modeling, for the avoidance of overfitting, and to ensure model reliability when it is performed on a new dataset.

Validation metrics involves the selection of appropriate metrics to evaluate model performance, such as accuracy, sensitivity, specificity, and mean squared error; the selected metrics should align with the specific experimental goals.

## 5. Raman Spectroscopy in Analysis of Carotenoids

In the case of an RS application, it is necessary to validate the obtained results via repetition of the experimental procedures under the same, similar, or somewhat altered experimental conditions, using the same objectives (samples), in addition to implementing methodological tools recommended by other research groups. In such cases, the detailed description of experimental conditions may be of crucial importance.

While reviewing reports on the use of Raman techniques and their experimental details in the characterization of carotenoids (Table 2), it can be noticed that some important parameters have not always been provided, including notes on the exposure and accumulation time, the number of recorded spectra, and resolution data (Table 2). In general, the principle of assigning the most expressed Raman bands to particular compounds based only on comparison with previously reported data in the literature and/or upon existing database values should be critically and carefully reconsidered. The Raman spectra bands originate from the vibration of particular functional groups reflecting the stretching movements between certain molecular bonds. Raman spectroscopy is a highly sensitive technique due to the use of specific instrumentation requiring constant and stable external conditions. Thus, the differences in spectra, i.e., the positions of particular bands (known as blue and red shift), occur even when using the same samples and the same instruments. Considering band-shifting phenomena, it is always better to assign compounds to classes by use of target functional group(s) rather than individual compounds. The spectral databases provided by RS manufacturers could be useful in studies of simple matrixes (composed of one to a few individual molecules). In the case of complex matrix systems, such as foods or plant extracts containing hundreds of different metabolites, the existing databases seem inapplicable. The same is true for the carotenoid mixtures naturally occurring in fresh, dry and extracted materials obtained from foods and other biological sources. In addition, the number of spectra used in classification and discrimination analyses is neither provided nor sufficient (Table 2). In general, there is no number of Raman spectra that is strictly recommended as sufficient for accurate RS observation, but the following equation is recommended to determine the optimal representative number of spectra:

$$n=\frac{\left(s_{1}^{2}+s_{2}^{2}\right)\cdot {\left(z_{1-\alpha }+z_{\beta }\right)}^{2}}{\delta^{2}}$$

Here, the following pertains:

- *n* is the required sample size for each group of studied samples/specimens;
- $s_{1}^{2}$ and $s_{2}^{2}$ are the variances within each group;
- $z_{1-\alpha }$ is the quantil of the standard normal distribution at the probability level 1−α, for a given significance level α;
- $z_{\beta }$ is the quantil of the standard normal distribution at the probability level β for achieving a power of 1 − *β*;
- δ is the minimum effect size one wants to detect.

Once the Raman spectra are collected, they should be divided into the training and test datasets according to the cross-validation method [66]. There is no exact rule on the number of training/test spectra, but the most accepted approximations are 75–80% and 20–25% for the training and test sets, respectively [66]. In the case of already-reported carotenoid studies, the total number of processed spectra is usually not provided, particularly as regards the number of spectra used in the training and test datasets (Table 2). The statistical processing of carotenoids’ Raman spectra (chemometrics) has mostly been performed using complex multivariate data analysis, which involves pre-processing and modeling procedures. In pre-processing, standard algorithms for the transformation of spectra are applied, while for data reduction, PCA is the most widely used method. The supervised classification models, such as PLS-DA and PLSR, are the most widely used discrimination and regression tools in carotenoids studies (Table 1). External data validation is absolutely necessary to ensure repeatability and good prediction model performances. Possibly because of the complex and time-consuming chemometrics procedure, external validation is not performed on RS analyses of carotenoids (Table 1). The error calculations used for discrimination and quantification models (RMSE) in RS-based carotenoids research are regularly reported (Table 1).

## 6. Carotenoids Quantification Modeling: From Starting Trials to Current Approaches

Forrest and Vilcins (1979) [67] were among the first to quantify β-carotene and lutein in plant material (tobacco leaves) by RS in combination with simple linear regression. The intensity of the characteristic bands was used as an independent variable (1160 and 1530 cm^−1^). At the beginning of the 21st century, Bhosale et al. (2004) [68] proposed RS as a tool to quantify carotenoids in many fruits and vegetables, such as tomatoes, carrots, spinach, cherries, oranges, etc. The results show that there was a high positive correlation between total carotenoids measured by HPLC and RS using a simple linear regression model (the independent variable was the band at 1525 cm^−1^). Nowadays, the technological development of computers allows the use of more complex multivariate regression models such as PLSR, SVMR, MLR, etc.

Recent achievements in the quantification of RS carotenoids have highlighted the importance of developing a chemometric transfer model capable of incorporating a transformation function that harmonizes the differences between multiple data sets (different samples, different acquisition times, etc.) to achieve better repeatability and reliability in RS quantification modeling (e.g., procrustes analysis, piecewise direct standardization, or wrapping method [63]).

The quantification of carotenoids is much more difficult than the identification of the dominant carotenoid molecules in a sample. To achieve the desired (stable, repeatable and accurate) model for the determination of the expected total quantity of carotenoids and/or individual carotenoids by RS, the following points must be considered: (a) The standard analytical methods for the determination of total and individual carotenoids (e.g., HPLC, HPTLC) should be simultaneously performed on the same material, serving as a key correction factor; (b) a series of pure carotenoids (chemical standards) and their mixtures should be prepared at concentrations corresponding to the concentrations determined in a sample by the standard analytical method, and exposed to RS measurements. Such a procedure allows the highest positioning probability of individual and mixed carotenoids in the Raman spectra.

There are some critical points to consider in the quantitative analysis of carotenoids (and other plant metabolites), as follows:

- Laser power and environmental (i.e., external) conditions should be stable and very similar during continuous RS measurements. As recommended, dividing the spectra into training and test datasets for the calibration and validation of the model is preferable;
- Recording the same samples in different terms (days, time-scale) for external validation is preferable so as to minimize the sensitivity of the Raman instrumentation. Upon obtaining results, it will be obvious whether the performed model is adequate or not. Testing different Raman spectra traits/variables (i.e., the height of the observed band, the area under the band, height/area band ratio, etc.) will provide the best matching with analytical data regarding the concentration of the analyte;
- Raman spectra are often shifted due to spectral variations that mostly originate from the sample, but could be also caused by external factors, which should be minimized during RS measurements.

**Figure 7:**
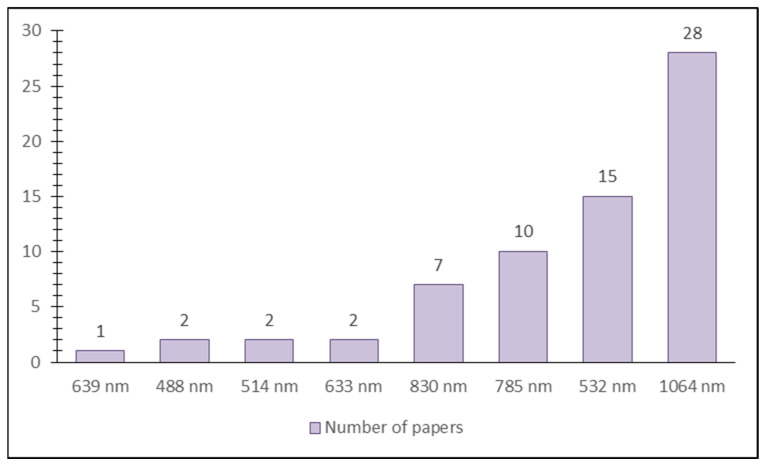
Lasers used in carotenoids analyses according available data.

Most of the papers focused on the qualitative analysis of carotenoids (43 out of 54), while quantitative analysis was conducted in 15 papers. However, external validation was not applied in any of them. RMSE (Root Mean Square Error) was calculated in 12 of 17 papers where it was applicable (Table 1). When it comes to the Raman scattering technique, in 61 experiments, a single-point approach was used, while image analysis was performed in only 4 (Table 2). The most commonly used laser was 1064 nm, while the least used was 488 nm (Figure 7 and Table 2).

**Figure 8:**
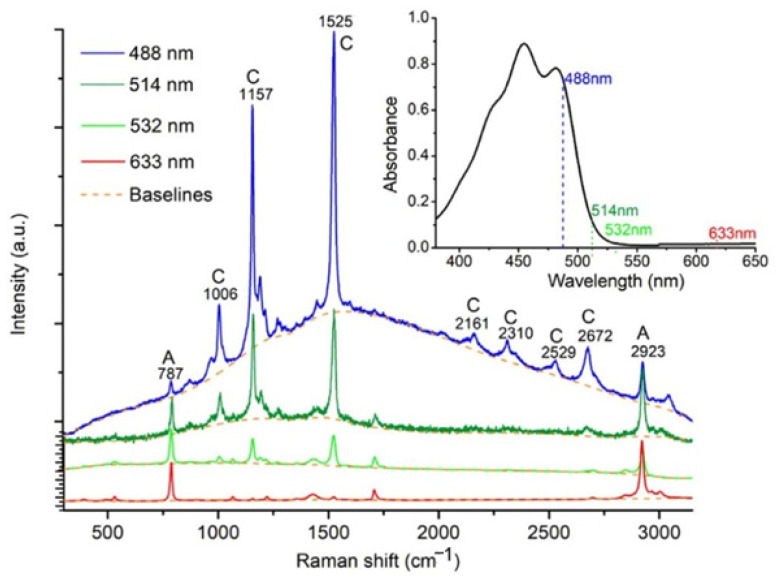
Raman spectra of β-carotene solution excited at 488, 514, 532 and 633 nm. The orange dashed curves represent the baselines for Raman spectra. The numbers with letter ‘C’ represent Raman peaks of β-carotene, while the numbers with ‘A’ represent the peaks originating from acetone molecules. The graphical presentation was taken from Lu et al. (2018) [71] with the authors’ permission.

The selection of a proper excitation wavelength is one of the most important steps affecting the acquisition time, spatial resolution, background fluorescence, etc. Generally, the selection of the laser depends on the traits of the studied sample. The choice of the laser to be used for analyzing carotenoids includes some important variables, such as the physical and chemical characteristics of a particular carotenoid compound, the traits of the sample matrix and the experimental goals. There are a lot of advantages and disadvantages to using different wavelengths [41,69,70,71,72]. Lu et al. (2018) [71] measured the ratio between carotenoids’ Raman bands and the background intensity, revealing that the 532 nm wavelength laser was the optimal Raman pump laser, with moderate Raman resonance enhancement due to reduced fluorescence and self-absorption, although the 488 nm and 514 nm pumps seemed at first to be better in imparting stronger Raman resonance enhancement (Figure 8). The 532 nm excitation will be useful when applying resonance Raman spectroscopy to investigate biological molecules in tissue. Comparing 780, 830 and 1064 nm, the 1064 nm laser was shown to provide more information and suppress the fluorescence. However, in this case, higher energy is needed, which consequently can cause damage to the biological material. To prevent such effects, some special sample preparation methods should be considered, namely, the freezing of the studied material [69]. More recently, various methods of fluorescence suppression have emerged, including time domain, frequency domain, wavelength domain and computational methods, as described in detail by Wei et al. (2015) [73].

The spectral resolution is one more parameter that can affect the informativeness of the spectrum. According to the literature, the resolution range of Raman spectra is from 0.1 to 15 cm^−1^, but the most frequent was between 3 and 4 cm^−1^ (Table 2).

A high spectral resolution is not required if only the determination of the presence of carotenoids in the sample is the aim of the analysis. However, to reveal precise details and to obtain subtle information from Raman spectra, especially regarding complex carotenoid samples (multi-carotenoid matrix), a much better spectral resolution is needed. Since different carotenoids have very similar structures, their Raman spectra are also similar. Therefore, in the case of low spectral resolution, the identification of different carotenoids present in complex mixture samples is not possible due to the closely spaced characteristic carotenoid bands. The spectral resolution depends directly on spectrometer focal length, grating, and the width of the entrance slit [74]. The longer the focal length is, the higher the spectral resolution that will be obtained. There are a lot of gratings, such as 300 g/mm, 600 g/mm, 1200g/mm, 1800 g/mm, and 2400 g/mm, as well as gratings with higher numbers of grooves per millimeter, which can also be used in analyses, allowing low spectral resolutions. Different gratings provide different spectral ranges. For example, with increasing groove density, better spectral resolutions are obtained, but at the same time, the spectral range will be narrower. In the case of carotenoids, the spectral range of any of the listed gratings will be suitable, but the correct choice depends on the experimental goal. Furthermore, the spectral resolution depends on the width of the entrance slit as well. When the laser light interacts with the sample molecules, the scattered light is focused on the entrance slit; it then passes to a collimating mirror, and after that towards the diffraction grating. Thus, the narrower the slit, the better resolution achieved. The slit regulates the amount of light that can pass through and reach the detector [75]. In this case, a somewhat better resolution can be obtained, but on the other hand, a smaller intensity of bands and a reduced SNR (signal to noise ratio) occur. Other important factors that can affect spectral resolution include detector size (a smaller pixel size corresponds to a higher spectral resolution) and laser wavelength (increasing the laser wavelength from UV to visible to IR will provide a better spectral resolution). These three parameters, along with focal length and grating, can be used in many different combinations to determine the highest spectral resolution that can be obtained with a particular Raman instrument. Indeed, there are some limitations affecting each of the parameters, with all these factors restricting the attainment of the highest possible spectral resolution in theoretical considerations.

**Table 1:** Review of statistical models applied in analyses of carotenoids.

Species	Type of Analysis	Aim of Investigation	Training and Test Sets	Applied Pre-Processing	Applied Models	External Validation (Yes/No)	RMSE (Yes/No)	Conclusion	References
Allium (*Allium* sp.)	QLT	Qualitative and classification analysis of different Allium species.	No	Baseline correction, normalization, PCA	PCA	No	NA	The analysis of the Raman spectra, combined with PCA, provided clear differences in the chemical profiles of the different *Allium* species samples.	[76]
Apple (*Malus domestica* L.)	QLT	Potential in monitoring fruit juice production.	NA	Baseline correction	NA	NA	NA	Raman spectroscopy can be used for direct online monitoring of carotenoids in fruit juice samples	[77]
Apricot (*Prunus armeniaca* L.)	QLT	Analyzing carotenoids in situ.	NA	No	NA	NA	NA	It has been found that FT-Raman spectroscopy can be successfully applied for the identification of carotenoids directly in the plant tissue without any preliminary sample preparation.	[48]
Apricot (*Prunus armeniaca* L.)	QLT	Potential in monitoring fruit juice production.	NA	Baseline correction	NA	NA	NA	Raman spectroscopy can be used for direct online monitoring of carotenoids in fruit juice samples.	[77]
Cabbage (*Brassica oleraceavar*. capitata)	QLT	Application of Raman spectroscopy to evaluate carotenoid composition in different green and red fruits, vegetables and spices.	NA	NA	NA	NA	NA	Raman spectroscopy can be used in carotenoids analysis of cabbage where β-carotene is detected.	[78]
Canabis/hemp (*Cannabis sativa* L.)	QLT and DIS	Discrimination between cannabis and hemp.	No	Baseline correction, SNV, first derivative.	OPLS-DA	No	NA	Raman spectroscopy can be used in discrimination between hemp and cannabis with 100% accuracy.	[79]
Carrot (*Daucus carota* L.)	QNT	Quantification of carotenoids in carrots using Raman spectroscopy.	No	Baseline correction and PCA	PCR, PLSR and LS-SVM	No	Yes	Models showed better prediction when characteristic bands were used (at 1006, 1154 I 1518 cm^−1^) compared to when the whole spectra were used. PLS showed the best results (R^2^ = 0.927–0.959 RMSE 4.66 mg/kg).	[21]
Carrot (*Daucus carota* L.)	QLT	Three complementary instrumental methods (Raman imaging, AFM and SNOM) were applied to reveal differences in carotenoid crystals accumulating in carrot cells.	NA	No	NA	NA	NA	Raman imaging using two excitation lines revealed different compositions of crystals and have provided evidence that crystals of planar structure, i.e., needle-like and rhomboidal, do not differ in their constitution, and they are composed of β-carotene and α-carotene, implying the crystals in the carrot root are the same.	[80]
Carrot (*Daucus carota* L.)	QLT	Monitoring biosynthesis and accumulation of carotenoids during development of carrot. Raman spectroscopy was employed to investigate the evolutions of carotenoids’ development and the influence of light in different stages of growth.	No	Baseline correction	NA	NA	NA	Raman spectroscopy can be used in the monitoring of the biosynthesis, transformation and accumulation of carotenoids during the growth of carrot without any pretreatment of the sample.	[81]
Carrot (*Daucus carota* L.)	QLT, QNT and DIS	Quantification of carotenoids in carrot as well as discrimination between different carrot genotypes.	Cross-validation	Second derivative for quantification	PLSR PCA	No	Yes	FT-Raman spectroscopy can be used for the quantification of carotenoids in carrot with regression coefficients over 0.96. PCA showed potential use in the discrimination of different carrot varieties.	[61]
Carrot (*Daucus carota* L.) 31 carrot lines	QNT	Quantification of carotenoids in carrot samples.	Yes	Smoothing and baseline correction, SNV, MSC, PCA	MLR, PLSR	No	Yes	Raman spectroscopy can be used for carotenoid quantification and discrimination of selected carrot samples. The regression model applied showed a high regression coefficient (R^2^ = 0.88 and 0.85 for calibration and validation, respectively).	[59]
Carrot red, yellow (*Daucus carrota* L.)	QLT	Application of Raman spectroscopy to evaluate carotenoid composition in different green and red fruits, vegetables and spices.	NA	NA	NA	NA	NA	Raman spectroscopy can be used in carotenoids analysis of red and yellow carrot where β-carotene and lutein were detected.	[78]
Carrot red, yellow (*Daucus carrota* L.)	QNT	Qunatification of total carotenoid content in carrot tissues.	No	Baseline correction and normalization	PLS-R	No	Yes	Results showed that Raman spectroscopy in combination with PLS-R can be used in total carotenoids quantification in carrot (R^2^ = 0.86).	[82]
Chinese chives (*Allium odorum* L.)	QLT	Application of Raman spectroscopy to evaluate carotenoid composition in different green and red fruits, vegetables and spices.	NA	NA	NA	NA	NA	Raman spectroscopy can be used in carotenoids analysis of Chinese chives where β-carotene was detected.	[78]
Chive (*Allium schoenoprasum* L.)	QLT	Application of Raman spectroscopy to evaluate carotenoid composition in different green and red fruits, vegetables and spices.	NA	NA	NA	NA	NA	Raman spectroscopy can be used in carotenoids analysis of chive where β-carotene was detected.	[78]
*Citrus* sp.	QLT	To investigate the relationship between freshness and obtained Raman spectra.	NA	Normalization	NA	NA	NA	Raman spectroscopy demonstrated that the intensity of the carotenoid (pre)-resonance Raman signal is an excellent indicator of the intact citrus freshness, thus introducing objective criteria of appreciation and quality control.	[83]
Corn (*Zea mays* L.)	QLT	Discrimination of different maize varieties.	No	No	PLS-DA	No	NA	Raman spectroscopy in combination with PLS-DA can be used for the discrimination of different maize varieties (precision 88–99%).	[84]
Corn (*Zea mays* L.)	QLT	Application of hand-held Raman spectrometer in combination with chemometrics to discriminate healthy and diseased maize kernels.	No	Baseline correction, normalization, PCA	OPLS-DA	No	NA	Raman spectroscopy coupled with OPL-DA discrimination model showed 100% precision.	[16]
Different vegetables	QNT	Determination of carotenoids in vegetables before and during boiling.	No	No	No	No	No	Raman spectroscopy can be used for the monitoring of changing of carotenoids contents during boiling of carotenoid-rich vegetables.	[85]
Dog rose (*Rosa canina* L.)	QLT and DIS	To discriminate different rosehip samples using Raman spectroscopy and chemometrics.	No	Baseline correction and normalization	PCA	No	NA	The results confirm the potential use of Raman spectroscopy as a fast and sophisticated method for obtaining detailed information concerning the spatial distribution of plant metabolites in the studied rosehips.	[86]
Dog rose (*Rosa canina* L.)	QLT	Application of Raman spectroscopy to evaluate carotenoid composition in different green and red fruits, vegetables and spices.	NA	NA	NA	NA	NA	Raman spectroscopy can be used in carotenoids analysis of dog rose where carotenoids were detected.	[78]
Extravirgin olive oil—EVOO	QNT	Quantification of carotenoids in EVOO.	No	Nonlinear curve fit based on Levenberg–Marquardt minimization algorithm	Simple linear regression	NE	No	Raman spectroscopy can be used to quantify the lutein/β carotene ratio in EVOOs from a single drop of oil.	[58]
Falso guarana (*Bunchosia glandulifera* (Jacq.) Kunth	QNT	To evaluate the carotenoids content of processed *Bunchosia glandulifera.*	No	PCA	PLSR	No	Yes	Raman spectroscopy can be used for the determination of carotenoids without sample preparation. There is a correlation between the area of characteristic bands and carotenoids content in the samples.	[60]
Garlic (*Allium sativum* L.)	QLT	Application of Raman spectroscopy to evaluate carotenoid composition in different green and red fruits, vegetables and spices.	NA	NA	NA	NA	NA	Raman spectroscopy can be used in carotenoids analysis of garlic where β-carotene and lutein were detected.	[78]
Grape (*Vitis vinifera* L.)	QLT	Application of Raman spectroscopy to evaluate carotenoid composition in different green and red fruits, vegetables and spices.	NA	NA	NA	NA	NA	Raman spectroscopy can be used in carotenoids analysis of grape where zeaxhantine, β-carotene and lutein were detected.	[78]
Lettuce butter (*Lactuca sativa* var. *capitata*)	QLT	Application of Raman spectroscopy to evaluate carotenoid composition in different green and red fruits, vegetables and spices.	NA	NA	NA	NA	NA	Raman spectroscopy can be used in carotenoids analysis of butter lettuce where β-carotene was detected.	[78]
Mandarine orange (*Citrus reticulata* Blanco)	QLT	Application of Raman spectroscopy in combination with pattern recognition approach to classification of citrus fruit.	No	Baseline correction and normalization	PCA, HCA	No	NA	It is concluded that Raman spectroscopy in combination with PCA and HCA can be used as a tool for quality assessment. According to PCA, two groups were obtained, while HCA showed slightly different results.	[87]
Mango (*Mangífera indica* L.)	QLT	Application of Raman spectroscopy to evaluate carotenoid composition in different green and red fruits, vegetables and spices.	NA	NA	NA	NA	NA	Raman spectroscopy can be used in the carotenoids analysis of mango, where β-carotene was detected.	[78]
Nectarine (*Prunus perica* L. var. *nucipersica*)	QLT	Application of Raman spectroscopy to evaluate carotenoid composition in different green and red fruits, vegetables and spices.	NA	NA	NA	NA	NA	Raman spectroscopy can be used in carotenoids analysis of nectarine where β-Cryptoxanthin was detected.	[78]
Orange (*Citrus* × *aurantium* L.)	QLT	Application of Raman spectroscopy to evaluate carotenoid composition in different green and red fruits, vegetables and spices.	NA	NA	NA	NA	NA	Raman spectroscopy can be used in carotenoids analysis of orange where β-carotene and β-Cryptoxanthin were detected.	[78]
Palm oil	QLT	Determination of ripeness level of oil palm fruitlets.	Yes—fivefold validation scheme	Baseline correction, smoothing	CRT, SVM, KNN	No	NA	Raman spectroscopy can be used to assess the ripeness level of oil palm fruitlets using carotenoids information and classification models. The best model was KNN (100% accuracy).	[88]
Papaya (*Carica papaya* L.)	QLT	Application of Raman spectroscopy to evaluate carotenoid composition in different green and red fruits, vegetables and spices.	NA	NA	NA	NA	NA	Raman spectroscopy can be used in carotenoids analysis of papaya pulp where lycopene, β-Cryptoxanthin, and β-carotene were detected.	[78]
Corn (*Zea mays* L.), Pumpkin (*Cucurbita pepo* L.), Paprika (*Capsicum annuum* L.), Watermelon (*Citrullus lanatus* L.), Apricot *Prunus armeniaca* L.), achiote tree (*Bixa orellana* L.), Nectarine(*Prunus perica* L. var. *nucipersica*), French bean (*Phaseolus vulgaris* L.), Saffron (*Crocus sativus* L.), Broccoli (*Brassica oleracea var. italica)*	QLT	To demonstrate potential of FT-Raman for in situ analysis of carotenoids-rich samples.	NA	No	NA	NA	NA	The results show that Raman spectroscopy can be used in the investigation of the *cis-trans* isomerization of carotenoids ruing processing. Besides this, 2-D Raman imaging presents the possibility of evaluating the distribution of carotenoids in plant tissue.	[48]
Peanut (*Arachis hypogaea* L.)	QLT and DIS	Discrimination of different peanut genotypes.	Cross-validation	Baseline correction	OPLS-DA	No	NA	Raman spectroscopy can be used for accurate identification of peanuts based on spectroscopic signatures of their leaves and seeds. OPLS-DA results show that peanut seeds can be identified with, on average, 95% accuracy, whereas the accuracy of Raman-based identification of leaves is, on average, 80%.	[89]
Pepper (*Capsicum annuum* L.)	QLT, QNT and DIS	Application of Raman spectroscopy to discriminate adulterated samples of paprika powder from the original one. Besides this, the quantification of ASTA color and Sundan I (adulterant).	Yes, 66 traiining, 22 test sets	Polynomial fitting	PLSR, PCA za klasifikaciju	NE	Yes	Raman spectroscopy showed two groups in the PCA score plot. Quantification displayed high regression coefficient for both ASTA and Sudan I concentrations (0.945 and 0.982, respectively).	[82]
Pepper (*Capsicum annuum* L.)	QLT, DIS	Classification of different paprika varieties at their final maturity stages.	Yes	Baseline correction, normalization, smoothing, PCA	PCA-LDA, PLS-DA, QDA	No	NA	Raman spectroscopy in combination with chemometrics can be used as a tool for the discrimination of different paprika varieties. The best classification results were acquired using QDA (100%).	[90]
Pepper (*Capsicum annuum* L.)	QLT and QNT	Qualitative and quantitative changes of carotenoids in paprika samples during plant development or under stress condition. Spatial distribution of carotenoids (Raman mapping).	No	Baseline correction	No	No	No	Raman spectroscopy can be used in qualitative and quantitative carotenoid analyses in paprika samples. Besides this, changes in these parameters during development can be monitored using Raman imaging where the distributions and relative amounts of carotenoids are presented.	[47]
Pepper (*Capsicum annuum* L.)	QLT, DIS	Assessment of red pepper ripening phases and carotenoids Accumulation.	Yes	Baseline correction, normalization, PCA	PCA-LDA, PLS-DA, SIMCA	No	No	Raman spectroscopy coupled with chemometrics can be used in determination of ripening phase. SIMCA showed the best classification accuracy (100%).	[56]
Pepper (*Capsicum annuum* L.)	QLT and DIS	Application of Raman spectroscopy in monitoring ripeness of hot paprika. PCA was applied to discriminate paprika fruits in different maturity stages.	No	SNV	PCA	No	NA	Raman spectroscopy can be used in the monitoring of ripeness of hot paprika as well as to discriminate different maturity stages.	[41]
Potato (*Solanum tuberosum* L.)	QLT	Identification of nine different potato varieties as well as determination of geographic origin of potato.	No	Third-order derivative	PLS-DA	No	NA	Raman spectroscopy can be used to identify nine different potato varieties, as well as to determine the origin of their cultivation.	[91]
Pumpkin (*Cucurbita pepo* L.)	QLT	Application of Raman spectroscopy to evaluate carotenoid composition in different green and red fruits, vegetables and spices.	NA	NA	NA	NA	NA	Raman spectroscopy can be used in carotenoids analysis of pumpkin where β-carotene was detected.	[78]
Purple onion (*Allium cepa* L.)	QLT	Application of Raman spectroscopy to evaluate carotenoid composition in different green and red fruits, vegetables and spices.	NA	NA	NA	NA	NA	Raman spectroscopy can be used in carotenoids analysis of purple onion where β-carotene was detected.	[78]
Rice (*Oryza sativa* L.)	QLT and DIS	Application of Raman spectroscopy to evaluate abiotic stresses.	No	Baseline correction, smoothing	PLS-DA	No	NA	Raman spectroscopy in combination with PLS-DA can be used in the discrimination of different stage of stresses. The accuracy of the applied model depends on the stage of stress (lower accuracy was obtained in samples with low stress and vice versa).	[79]
Sweet potato (*Ipomoea batatas* (L.) Lam.)	QNT	Quantitative analysis of carotenoids in processed sweet potato.	No	SNV, PCA	PLSR	No	Yes	Raman spectroscopy coupled with chemometrics can be used to quantify carotenoids in real time.	[59]
Tea leaves (*Camellia sinensis* L.)	QNT	In situ quantitative visualization of chlorophyll and carotenoid with calibration transfer model.	No	MSC, WT, SNV, RCF and airPLS.	PLSR	Yes, to second instrument	Yes	It can be concluded that Raman spectroscopy was applicable for in situ, non-destructive and rapid quantitative detection and imaging of photosynthetic pigment concentration in tea leaves, and the spectral detection model established based on the laboratory Raman spectrometer can be applied to a portable field spectrometer for quantitatively imaging the foliar pigments.	[92]
Tomato (*Lycopersicon esculentum* L.)	QNT	Quantification of carotenoids.	Cross-validation	Baseline correction	PLSR	No	No	The 785 nm laser has the most suitable excitation wavelength to perform a quantitative analysis of carotenoid concentrations in tomatoes.	[20]
Tomato (*Lycopersicon esculentum* L.)	QLT, QNT and DIS	To develop models for the quantification of carotenoids and discrimination of analyzed samples.	Cross-validation	Mean centering and smoothing, PCA	SIMCA, ANN, PLSR	No	Yes	SIMCA classification accuracy 93%, ANN 100%. PLSR and ANN for quantification regression coefficients over 0.9.	[55]
Tomato (*Lycopersicon esculentum* L.)	QLT	Monitoring carotenoids’ biosynthesis in situ during fruit development.	NA	Baseline correction	NA	NA	NA	Raman spectroscopy equipped with 1064 and 785 nm lasers and DFT calculations can be used for monitoring the main carotenoids during the ripening process.	[40]
Tomato (*Lycopersicon esculentum* L.)	QLT, QNT and DIS	It was found that Raman spectroscopy with a short exposure time can be used in the quantitative and discriminative analysis of carotenoids in intact tomato.	Yes, 40 for training and 20 for test set	Baseline correction	PLSR and PLS-DA	No	Yes	The results show that longer exposure times provide better R^2^ values. Accordingly, when the exposure time was 10 s and 0.7 s, R^2^ was 0.87 and 0.69, respectively. When it comes to discrimination, there were no significant differences depending on exposure time.	[20]
Tomato (*Lycopersicon esculentum* L.)	QLT	To evaluate the fruit quality of three traditional tomato genotypes with different colors from the Balkans.	NA	Normalization	PCA	No	NA	Carotenoid information obtained by Raman spectroscopy can be used in discrimination of different tomato varieties.	[93]
Tomato (*Lycopersicon esculentum* L.)	QNT	Quantification of lycopene and β-carotene in tomato fruits and related products.	Cross-validation	Baseline correction and normalization	PLSR	No	Yes	Results show that Raman spectroscopy can be used in quantification of lycopene and β-carotene. Accordingly, R^2^ was 0.91 and 0.89 for lycopene and β-carotene, respectively.	[46]
Tomato (*Lycopersicon esculentum* L.)	QLT, DIS and QNT	Does exposure time affect the precision of quantitative and discrimination analysis?	Yes, 40 for training and 20 for validation.	Mean centering	PLSR and PLS-DA	No	Yes	When it comes to quantification analysis, the best results were obtained when 10 s integration time was applied, and the worst when 0.7 s was applied. On the other hand, in the PLS-DA result, integration time did not affect precision.	[21]
Watermelon (*Citrullus lanatus* L.)	QLT	Determination of maturity stage of watermelon.	NA	Baseline correction	PLS-DA	No	Yes	Raman spectroscopy can be used to determinate the stage of maturity of watermelon.	[39]
Wheat (*Triticum aestivum* L.)	QLT and DIS	Discrimination of healthy and infected wheat.	No	Baseline correction	PLS-DA	No	NA	Raman spectroscopy can be used to differentiate between healthy wheat and wheat infected with viruses.	[57]

No—no data; NA—not applicable; QLT—qualitative analysis; QNT—quantitative analysis; DIS—discriminative analysis.

**Table 2:** Raman instrumentation parameters, spectral regions and characteristic bands in carotenoids analyses.

Species	Raman Scattering Technique	Laser Wavelength	Laser Power	Exposure Time	Accumulation Time	Grating	Number of Recorded Spectra	Objective	Spectral Resolution	Recorded Spectral Range Used in Analysis	Carotenoid Band Position	Reference
*Allium* sp.	point scan	532 nm	No	10 s	15	600 g/mm	No	-	3 cm^−1^	No	1527, 1153, 1002 cm^−1^	[76]
Apple (*Malus domestica* L.)	mapping	633 nm	17 mW	60–300 s	No	1800 g/mm	No	50x	4 cm^−1^	0–2000 cm^−1^	1008, 1159 and 1520 cm^−1^	[77]
Apricot (*Prunus armeniaca* L.)	point-scan	1064 nm	300 mW	No	No	No	512	No	4 cm^−1^	100–4000 cm^−1^	1524, 1156, 1003 cm^−1^	[48]
Apricot (*Prunus armeniaca* L.)	mapping	633 nm	17 mW	60–300 s	No	1800 g/mm	No	50×	4 cm^−1^	0–2000 cm^−1^	1520, 1159, 1008 cm^−1^	[77]
Cabbage (*Brassica oleracea var. capitata*)	point scan	1064 nm	70–300 mW	No	3000	No	No	No	1–3 cm^−1^	900–2000 cm^−1^	1517, 1158, 1008 cm^−1^	[78]
Canabis/hemp (*Cannabis sativa* L.)	point scan	831 nm	495 mW	10 s	No		20–23	No	15 cm^−1^	701–1700 cm^−1^	1527, 1155, 1000 cm^−1^	[79]
Carrot (*Daucus carota* L.)	Point-scan	785 nm	300 mW	10 s	2	No	No	No	6 cm^−1^	100–3200 cm^−1^	1006, 1154, 1518 cm^−1^	[21]
Carrot (*Daucus carota* L.)	imaging	532 nm	20 mW	0.1 s	No	No	No	No	5–6 steps/1 μm	No	1007–1010; 1159–1160; 1519–1523 cm^−1^	[80]
Carrot (*Daucus carota* L.)	imaging	488 nm	10 mW	0.3 s	No	No	No	No	2 steps/1 μm	No	1007–1010; 1159–1160; 1519–1523 cm^−2^	[80]
Carrot (*Daucus carota* L.)	point scan	532 nm	0.4 mW	10 s	No	No	No	50x	0.1 cm^−1^	400–1800 cm^−1^	1519, 1156, 1008 cm^−1^	[81]
Carrot (*Daucus carota* L.)	point scan	1064 nm	500 mW	No	64	No	5	No	4 cm^−1^	total carotenoids—4000–3619 cm^−1^; 2481–1339 cm^−1^; 961–199 cm^−1^	1527, 1157, 1108 cm^−1^	[61]
Carrot (*Daucus carota* L.)	point scan	1064 nm	500 mW	No	64	No	5	No	4 cm^−1^	α-carotene—3621–3239 cm^−1^; 1721–959 cm^−1^	1527, 1157, 1108 cm^−2^	[61]
Carrot (*Daucus carota* L.)	point scan	1064 nm	500 mW	No	64	No	5	No	4 cm^−1^	β-carotene—4000–3619 cm^−1^; 1721–199 cm^−1^	1527, 1157, 1108 cm^−3^	[61]
Carrot (*Daucus carota* L.)	point scan	1064 nm	500 mW	No	64	No	5	No	4 cm^−1^	lutein—4000–3619 cm^−1^; 1721–959 cm^−1^; 581–199 cm^−1^	1527, 1157, 1108 cm^−4^	[61]
Carrot (*Daucus carota* L.)	point scan	785 nm	200 mW	2 s	No	No	No	No	No	100–1900 cm^−1^	1520, 1156, 1007 cm^−1^	[82]
Carrot (*Daucus carota* L.), 31 carrot lines	point scan	1064 nm	120 mW	3 min	100	No	101	No	4 cm^−1^	0–3500 cm^−1^;	1520, 1156, 1006 cm^−0^	[69]
Carrot (*Daucus carota* L.), 31 carrot lines	point scan	830 nm	271 mW	4 min	16	No	101	No	No	200–2300 cm^−1^;	1520, 1156, 1006 cm^−1^	[69]
Carrot (*Daucus carota* L.), 31 carrot lines	point scan	785 nm	No	4 min	16	No	101	No	No	200–2300 cm^−1^	1520, 1156, 1006 cm^−2^	[69]
Carrot red, yellow (*Daucus carrota* L.)	point scan	1064 nm	70–300 mW	No	3000	No	No	No	1–3 cm^−1^	900–2000 cm^−1^	1527, 1157, 1108 cm^−1^	[78]
Carrot red, yellow (*Daucus carrota* L.)	point scan	785 nm	400 mW	0.1 s	150	No	88	-	No	300–1800 cm^−1^	1521, 1157, 1107 cm^−1^	[82]
Carrot red, yellow (*Daucus carrota* L.)	point scan	785 nm	100 mW	No	No	No	64	No	13 cm^−1^	400–2300 cm^−1^	1001, 1156, 1515 cm^−0^	[41]
Carrot red, yellow (*Daucus carrota* L.)	point scan	639 nm	35 mW	No	No	No	64	No	7–10 cm^−1^	150–3150 cm^−1^	1001, 1156, 1515 cm^−1^	[41]
Carrot red, yellow (*Daucus carrota* L.)	point scan	1064 nm	900 mW	No	No	No	64	No	6 cm^−1^	100–3800 cm^−1^	1001, 1156, 1515 cm^−2^	[41]
Carrot red, yellow (*Daucus carrota* L.)	image	1064 nm	300 mW	3000 s	No	No	No	No	4 cm^−1^	100–4000 cm^−1^	1524, 1510 cm^−1^	[47]
Chinese chives (*Allium odorum* L.)	point scan	1064 nm	70–300 mW	No	3000	No	No	No	1–3 cm^−1^	900–2000 cm^−1^	1527, 1153, 1002 cm^−1^	[78]
Chive (*Allium schoenoprasum* L.)	point scan	1064 nm	70–300 mW	No	3000	No	No	No	1–3 cm^−1^	900–2000 cm^−1^	1527, 1158, 1006 cm^−1^	[78]
*Citrus* sp.	point scan	532 nm	Clementine-200 mW, mandarins and tangarins 20 mW	1 s	1	No	No	20×	0.5 cm^−1^	100–1800 cm^−1^	1520–1523, 1153–1155, 1003 cm^−1^	[83]
Corn (*Zea mays* L.)	point scan	830 nm	432 mW	1 s	No	No	Around 100 per variety	-	15 cm^−1^	400–1800 cm^−1^	1527, 1153 cm^−1^	[84]
Corn (*Zea mays* L.)	point scan	1064 nm	200 mW	10 s	No	No	-	No	No	400–1700 cm^−1^	1523, 1155, 1003 cm^−1^	[16]
Corn (*Zea mays* L.), Pumpkin (*Cucurbita pepo* L.), Pepper (*Capsicum annuum* L.), Watermelon (*Citrullus lanatus* L.), Apricot *Prunus armeniaca* L.), achiote tree (*Bixa orellana* L.), Nectarine (*Prunus perica* L. var. *nucipersica*), French bean (*Phaseolus vulgaris* L.), Saffron (*Crocus sativus* L.), Broccoli (*Brassica oleracea var. italica*)	point scan	1064 nm	Samples—300	No	No	No	No	No	No	800–1800 cm^−1^	Apricot-1526, 1156, 1000 cm^−1^ French bean—1522, 1157, 1005 cm^−1^; Corn-1522, 1157, 1005; Nectaraine-1527, 1157, 1005 cm^−1^; Pumpkin-1550–1600, 1450, 1360–1390, 1100, 950–980, 520–530; Tomato-1510, 1156, 1005 cm^−1^; Watermelon-1510, 1158, 1008 cm^−1^; Saffron-1536, 1165, 1020 cm^−1^; Broccoli-1524, 1157, 1005 cm^−1^; Carrot—1526, 1157, 1004 cm^−1^; Annatto—1518, 1154, 1011 cm^−1^	[48]
Different vegetables	point scan	488 nm	/	No	No	No	No	6	No	No	1525 cm^−1^	[85]
Dog rose (*Rosa canina* L.)	point scan	532 nm	No	10	10	1200 g/mm	No	No	3 cm^−1^	200–1800 cm^−1^	1513, 1153, 1003 cm^−1^	[86]
Dog rose (*Rosa canina* L.)	point scan	1064 nm	70–300 mW	No	3000	No	No	No	1–3 cm^−1^	900–2000 cm^−1^	1513, 1153, 1000 cm^−1^	[78]
Extravirgin olive oil EVOO	point scan	532 nm	5 mW	10 s	5	No	9	10×	No	750–1800 cm^−1^	1520, 1156 cm^−1^	[58]
Falso guarana (*Bunchosia glandulifera* (Jacq.) Kunth	point scan	532 nm	50 mW	10 s	3	1800 g/mm	6	No	No	400–2000 cm^−1^	1524, 1157, 1005 cm^−1^	[60]
Garlic (*Allium sativum* L.)	point scan	1064 nm	70–300 mW	No	3000	No	No	No	1–3 cm^−1^	900–2000 cm^−1^	1511, 1179, 1005 cm^−1^	[78]
Grape (*Vitis vinifera* L.)	point scan	1064 nm	70–300 mW	No	3000 scans	No	No	No	1–3 cm^−1^	900–2000 cm^−1^	1525, 1155, 1004 cm^−1^	[78]
Lettuce butter (*Lactuca sativa var capitata*)	point scan	1064 nm	70–300 mW	No	3000	No	No	No	1–3 cm^−1^	900–2000 cm^−1^	1517, 1158, 1008 cm^−1^	[78]
Mandarine orange (*Citrus reticulata* Blanco)	point scan	514 nm	20 mW	10	No	No	No	No	1.5 cm^−1^	100–3200 cm^−1^	1520, 1155, 1003 cm^−1^	[87]
Mango (*Mangífera indica* L.)	point scan	1064 nm	70–300 mW	No	3000	No	No	No	1–3 cm^−1^	900–2000 cm^−1^	1513, 1153, 1000 cm^−1^	[78]
Nectarine (*Prunus perica* L. var. *nucipersica*)	point scan	1064 nm	70–300 mW	No	3000	No	No	No	1–3 cm^−1^	900–2000 cm^−1^	1529, 1157, 1004 cm^−1^	[78]
Orange (*Citrus* × *aurantium* L.)	point scan	1064 nm	70–300 mW	No	3000	No	No	No	1–3 cm^−1^	900–2000 cm^−1^	1531, 1156, 1012 cm^−1^	[78]
Palm oil	point scan	532 nm	2 mW	3 s	3	900 g/mm	No	No	No	No	1000, 1150, 1515 cm^−1^	[88]
Papaya pulp (*Carica papaya* L.)	point scan	1064 nm	70–300 mW	No	3000	No	No	No	1–3 cm^−1^	900–2000 cm^−1^	1525, 1155, 1008 cm^−1^	[78]
Peanut (*Arachis hypogaea* L.)	point scan	830 nm	495 mW	1 s	No	No	No	No	No	400–1800 cm^−1^	1526, 1155, 1000 cm^−1^	[89]
Pepper (*Capsicum annuum* L.)	point scan	532 nm	20–25 mW	5 s	10	1200 g/mm	50	50×	3 cm^−1^	900–1800 cm^−1^	1511–1519, ~1149–1151,	[90]
Pepper (*Capsicum annuum* L.)	point scan	532 nm	20–25 mW	5 s	10	1200 g/mm	50	50×	3 cm^−1^	900–1800 cm^−1^	~998–1006 cm^−1^	[90]
Pepper (*Capsicum annuum* L.)	point scan	532 nm	20–25 mW	3 s	10	1200 g/mm	50	50×	3 cm^−1^	900–1800 cm^−1^	1003, 1151, 1514–1517 cm^−1^	[56]
Pumpkin (*Cucurbita pepo* L.)	point scan	1064 nm	70–300 mW	No	3000	No	No	No	1–3 cm^−1^	900–2000 cm^−1^	1527, 1157, 1108 cm^−1^	[78]
Purple onion (*Allium cepa* L.)	point scan	1064 nm	70–300 mW	No	3000	No	No	No	1–3 cm^−1^	900–2000 cm^−1^	1515, 1151, 1014 cm^−1^	[78]
Rice (*Oryza sativa* L)	point scan	830 nm	495 mW	1 s	No	No	No	No	No	600–1800 cm^−1^	1000, 1155, 1527 cm^−0^	[79]
Sweet orange (*Citrus* × *sinensis*)	point scan	514 nm	20 mW	10 s	No	No	No	No	1.5 cm^−1^	100–3200 cm^−1^	1520, 1155, 1003 cm−^1^	[87]
Sweet potato (*Ipomoea batatas* (L.) Lam.)	point scan	532 nm	50 mW	No	5	1800 g/mm	30	50x	No	250–2000 cm^−1^	1520, 1157, 1006 cm^−1^	[59]
Tea leaves (*Camellia sinensis* L.)	point scan	532 nm	50 mW	1 s	No	No	315	No	0.2 cm^−1^	792–1961 cm^−1^	1008, 1159, 1528 cm^−1^	[92]
Tomato (*Lycopersicon esculentum* L.)	point scan	532 nm	25 mW	0.2 s	1	No	No	No	1.45 cm^−1^	800–1800 cm^−1^	1006, 1156, 1518 cm^−1^	[20]
Tomato (*Lycopersicon esculentum* L.)	point scan	785 nm	176 mW	10 s	1	No	No	No	8 cm^−1^	800–1800 cm^−1^	1006, 1156, 1518 cm^−2^	[20]
Tomato (*Lycopersicon esculentum* L.)	point scan	1064 nm	490 mW	0.5 s	3	No	No	No	11 cm^−1^	800–1800 cm^−1^	1006, 1156, 1518 cm^−3^	[20]
Tomato (*Lycopersicon esculentum* L.)	point scan	1064 nm	/	10 s	30	No	No	No	8 cm^−1^	200–2500 cm^−1^	1007, 1158, 1520 cm^−1^	[55]
Tomato (*Lycopersicon esculentum* L.)	point scan	785 nm	176 mW	0.7 s and 10 s	1	No	No	No	1.4 cm^−1^	975–1615 cm^−1^	1156 cm^−1^	[21]
Tomato (*Lycopersicon esculentum* L.)	point scan	1064 nm	1064–150 mW	No	1024	No	No	No	4 cm^−1^	No	1519–1528, 1158, 1007 cm^−1^	[40]
Tomato (*Lycopersicon esculentum* L.)	point scan	785 nm	785–100 mW	10 s	No	No	No	50×	No	No	1519–1528, 1158, 1007 cm^−2^	[40]
Tomato (*Lycopersicon esculentum* L.)	point scan	785 nm	176 mW	10 and 0.7	No	No	60	-	1.45 cm^−1^	800–1800 cm^−1^ for discrimination model	1516, 1156, 1006 cm^−1^	[20]
Tomato (*Lycopersicon esculentum* L.)	point scan	785 nm	176 mW	10 and 0.7	No	No	60	-	1.45 cm^−1^	1615–975 cm^−1^ f or regression model	1516, 1156, 1006 cm^−2^	[20]
Tomato (*Lycopersicon esculentum* L.)	point scan	532 nm	/	5 s	5	1200 g/mm	No	50×	3 cm^−1^	200–1800 cm^−1^	1505–1515, 1148, 997 cm^−1^	[93]
Tomato (*Lycopersicon esculentum* L.)	point scan	1064 nm	150 mW	No	512	No	No	No	4 cm^−1^	100–4000 cm^−1^	1370, 965–960 cm^−1^	[46]
Watermelon (*Citrullus lanatus* L.)	point scan	830 nm	495 mW	1 s	No	No	20–43	No	No	400–2000 cm^−1^	1002, 1,156, 1,186, 1,217, and 1,525 cm^−1^	[39]
Wheat (*Triticum aestivum* L.)	point scan	830 nm	320 mW	1 s	No	No	5–10 per leaf	-	15 cm^−1^	400–1800 cm^−1^	1525, 1156, 1000 cm^−1^	[57]

No—no data.

## 7. New Raman Approaches in Food Carotenoids Analysis

### 7.1. Spatially Offset Raman Spectroscopy

Spatially Offset Raman Spectroscopy (SORS) is an advanced variant of Raman spectroscopy that enables the detection and analysis of chemical compositions beneath the surface of diffusely scattering materials. Unlike conventional Raman spectroscopy, which primarily collects signals from the surface or near-surface regions of a sample, SORS can probe deeper layers, making it particularly valuable in fields such as pharmaceuticals [94], biomedical diagnostics [95], and food and beverages [96].

The fundamental principle of SORS is based on the spatial separation between the incident laser beam and the collection point of the Raman scattered light. In conventional Raman spectroscopy, the laser excitation and the collection optics are typically aligned to focus on the same spatial point on the sample’s surface. In contrast, SORS involves deliberately offsetting the collection point from the illumination point. This spatial offset allows the collection of Raman signals that have scattered through subsurface layers, enabling the detection of materials that are otherwise obscured by the surface layer [97,98].

When the laser light interacts with a sample, photons are scattered in all directions. Photons that penetrate deeper into the material and are subsequently scattered back towards the surface can be collected by a detector positioned at a spatial offset from the laser spot. The Raman signals collected in this manner are enriched with information from beneath the surface, as photons that travel longer paths are more likely to contain subsurface contributions [97,98].

Qin et al. (2011) [99] applied SORS in the evaluation of the internal maturity of tomatoes. The results show that SORS can be used for detecting subsurface signals through outer pericarps up to 10 mm in thickness. The Raman peaks observed in mature green and breaker tomatoes suggest that carotenoids in the locular tissue can be detected using the SORS-SMA (self-modeling mixture analysis) method, as the green outer pericarps lack Raman-active carotenoids. This paper is the first to link SORS with carotenoids. Potential future applications include analyzing the surface layers of carotenoid-rich products like fruits and vegetables, as well as measuring carotenoids in encapsulated items or through transparent or semi-transparent packaging. This approach could allow for the quality assessment of packaged foods without opening them. However, SORS may reduce the signal-to-noise ratio and limit the depth of analysis, and the overlap of signals from surface and subsurface layers might require advanced data processing techniques for separation. In addition, the SORS application has only been applied in a few papers when it comes to carotenoids in plant material [99,100].

Since SORS can provide information of chemical composition through the depth of the fruit, it is the perfect instrument for analyzing the level of maturation of climacteric fruits such as tomatoes, avocados, red banana, apricot, peaches, etc. So, future investigations should be oriented toward the application of SORS in in situ analyses of the mentioned fruits. However, due to the increase in the signal/noise ratio, one should be careful in processing the results, and perform the chemometric analyses in the right way.

### 7.2. Coherent Anti-Stokes Raman Spectroscopy

Coherent Anti-Stokes Raman Spectroscopy (CARS) is an advanced vibrational spectroscopy technique that offers several advantages over conventional Raman spectroscopy, particularly in terms of sensitivity and the ability to probe molecular vibrations with high spatial and temporal resolution [101]. CARS is particularly advantageous for studying carotenoids due to their strong Raman-active vibrational modes, especially those associated with C=C stretching vibrations. The main advantage is high-sensitivity detection, whereby CARS can detect carotenoids at very low concentrations due to its enhanced sensitivity compared to conventional Raman techniques. This is important for analyzing trace amounts of carotenoids in complex food matrices or biological tissues. Improved SNR makes it easier to distinguish the carotenoid signal from background noise or fluorescence, which is often a challenge in spontaneous Raman spectroscopy.

When it comes to the imaging and mapping of carotenoids, CARS provides high spatial resolution, enabling the detailed imaging of carotenoid distribution within biological tissues or food products. This capability is particularly useful in visualizing the distribution of carotenoids in cells, tissues, or even whole organisms. The technique can be used to map the distribution of carotenoids in complex food products, such as fruits and vegetables, providing insights into the nutritional content and quality. For example, CARS can be used to visualize the distribution of beta-carotene in orange-fleshed sweet potato, carrot and mango [102].

CARS allows for the non-invasive imaging of carotenoids in living organisms, such as plants or animals. This is crucial for studying dynamic processes, like carotenoid biosynthesis, transport, and storage, in real time without harming the sample. Unlike some analytical techniques that require extensive sample preparation, CARS can be performed with minimal preparation, preserving the natural state of the sample. Dementjev and Kostkevičiene (2013) [103] applied CARS to visualize carotenoids in microalgae, providing high-resolution imaging, while Chen et al. (2017) [104] demonstrated that the CARS imaging system achieved an improvement of up to two orders of magnitude in both refresh time and measurement time. The realization of such achievements is enabled through the rapid-scanning of down-converted delay time in conjunction with the ultrafast acquisition of asynchronous optical geometry. This phase-controlled excitation circumvents the trade-off between high SNR and improved refresh rate by concentrating most of the tailored optical driver fields into a single Raman mode.

In the food industry, CARS can also be potentially used to monitor carotenoid content during processing and storage, ensuring that the nutritional and sensory qualities of the product are maintained. For example, it can be used to monitor the degradation of carotenoids during thermal processing or storage. CARS can help verify the authenticity of food products by analyzing the specific carotenoid profiles, which can be used to differentiate between varieties or detect adulteration. For instance, CARS can be used to ensure that a product labeled as “high in beta-carotene” actually contains the expected levels of this carotenoid. In plants, CARS can be used to study the biosynthesis pathways of carotenoids, providing insights into how these compounds are produced and stored in different parts of the plant. CARS can also be applied to study how carotenoids are absorbed and metabolized in the human body, offering potential applications in nutritional research and health diagnostics.

### 7.3. Stimulated Raman Spectroscopy

Stimulated Raman Spectroscopy (SRS) is an advanced form of Raman spectroscopy with enhanced detection sensitivity and imaging speed achieved by using two synchronized laser beams—a pump beam and a Stokes beam. When the frequency difference between these two beams matches the vibrational frequency of a molecular bond, it induces a stimulated Raman scattering process. This process results in an amplified Raman signal, allowing for the rapid and highly sensitive detection of molecular vibrations. Unlike traditional Raman spectroscopy, which relies on spontaneous scattering and thus typically yields weak signals, SRS generates a much stronger signal by stimulating the Raman process. This makes SRS particularly well-suited for high-speed, high-resolution imaging, and for studying complex biological samples.

Carotenoids, such as β-carotene, lycopene, and lutein, exhibit distinct vibrational modes that are well-suited for SRS detection. The strong enhancement of the Raman signal in SRS allows for the detection of carotenoids even at low concentrations, making it possible to study their distribution and concentration in biological tissues, foods, and supplements with high sensitivity.

SRS enables the label-free, non-destructive imaging of carotenoids in living cells and tissues. This is particularly valuable for studying carotenoid distribution in plant cells, where carotenoids play crucial roles in photosynthesis and photoprotection. The high spatial resolution of SRS allows researchers to visualize carotenoid-rich regions within cells, such as chloroplasts, and monitor changes in carotenoid content in response to environmental factors. Comparing with CARS, SRS has a linear dependence on concentration, which can be useful for the label-free quantification of carotenoids. Further, SNR is improved in SRS [105,106].

Some authors have applied SRS in plant analysis to investigate the distribution of plant epicuticular waxes and the presence of agrochemicals on leaves, cotton and maize [107], as well as to investigate carotenoids in photosynthetic light-harvesting processes [108,109]. However, according to some authors, it is still necessary to increase the consistency and reproducibility of CARS or SRS in order to make these techniques more suitable for the large-scale study of carotenoids [109,110].

In the food industry, SRS can be used to map the distribution of carotenoids in various food matrices, such as fruits, vegetables, and processed products. This is important for quality control, nutritional analysis, and the development of functional foods. SRS imaging can reveal how carotenoids are distributed within different food structures, affecting their bioavailability and nutritional value.

Carotenoids tend to aggregate, especially when present in high concentrations, which can affect their stability, absorption, and efficacy. SRS is particularly effective in studying these aggregates in situ, providing insights into how carotenoid aggregation influences their physical properties and interactions with other biomolecules, such as lipids and proteins. SRS can be used to study the interactions of carotenoids with other biological molecules, such as lipids and proteins, which is critical for understanding their bioavailability and function in biological systems. For instance, SRS can reveal how carotenoids interact with lipid membranes, influencing their incorporation and stability within these membranes.

## 8. Application of Artificial Intelligence (AI) in Carotenoid Analysis Using Raman Spectroscopy

AI can significantly enhance Raman microspectroscopy in carotenoid analysis, improving the precision, efficiency, and depth of analysis across single-point, 2D, and 3D imaging.

Despite its reputation for non-destructive chemical analysis, Raman spectroscopy frequently produces noisy, complex data that are difficult to handle. By lowering noise and increasing signal clarity, artificial intelligence (AI) techniques—in particular, machine learning and deep learning algorithms—have been included in the field to improve the quality of the spectra. For example, deep neural networks (DNN) are used to denoise spectra, which improves data reliability and makes them usable for precise further analyses, such as regression, classification or decomposition [111].

The interpretation of Raman spectra, especially the determination of chemical species, is one of the main fields wherein AI has had an impact. Raman spectra frequently have overlapping peaks that indicate several chemical vibrations, which makes manual interpretation laborious and susceptible to mistakes. Support vector machines (SVMs) [112], constant wavelet transform (CWT) [113] and convolutional neural networks (CNNs) [114], three AI-driven algorithms, can automate this procedure by quickly identifying peaks and linking them to certain chemical species. This has created new opportunities for quicker and more precise examinations of complicated samples with several different chemicals.

The decomposition of Raman spectra from a mixture presents a challenge, especially in cases of complex mixtures, such as food matrices. Non-Negative Matrix Factorization (NMF) is one of the AI-assisted algorithms used for spectral decomposition. The algorithm decomposes the original spectral data matrix into two smaller matrices—one representing the basis spectra (the underlying components) and the other representing their respective weights or contributions. This decomposition is helpful when analyzing mixtures of compounds or complex samples where individual spectra overlap. NMF can separate overlapping spectral peaks, making it easier to identify and quantify each component in the mixture. It is a popular tool used in chemometrics for analyzing the Raman spectra of samples with multiple constituents [115,116,117]. One more algorithm that can be used for spectral decomposition is Sparse Component Analysis (SCA). SCA is a non-linear technique that assumes that the observed spectrum can be represented by a sparse set of components. The goal of SCA is to find the most sparse set of components that can explain the data, which is particularly useful in situations where the spectral components are expected to be relatively sparse and isolated. This method is useful in relation to the Raman spectra of samples that contain only a few dominant components, allowing for clearer identification and quantification [118]. This algorithm can be used in the case of some simple food matrices.

## 9. Final Remarks

There is no doubt that RS will find wider application in the theoretical and applied disciplines dealing with biological materials. The continuous improvement of modern and sophisticated research equipment makes it possible to perform some advanced and innovative methods that currently still show certain limitations. Raman spectroscopy is an evolving technique that is expected to find application in food science and food quality control on an industrial scale. Raman spectroscopy is based on the inelastic scattering of photons, which reflects the stretching vibrations of characteristic molecular bonds, especially double and triple bonds, as well as various functional groups, in a single analyte or in mixtures of analytes. Raman spectroscopy makes it possible to obtain immediate information that actually represents a fingerprint of the material under investigation. The obvious superiority of RS over standard analytical methods (efficiency, ability to obtain a large number of spectra in a very short time, non-preparative, environmentally friendly, waste-free and user-friendly technique) should not be taken for granted, especially when observing natural products, food and other biological materials with extremely complex chemical compositions. In the present work, we have tried to highlight the main problems and limitations of using RS in the study of nutritionally valuable plant pigments—carotenoids, which serve as model molecules. Most of the reports have addressed the presence of total or dominant individual carotenoids in carotenoids-rich samples using the single-point Raman approach. Another group of studies has focused on the distribution of carotenoids in various biological (fruits, vegetables, cereals, bacteria, fish, egg yolk, etc.) and non-biological materials (synthetic carotenoids, cosmetic preparations, dietary supplements, pharmaceuticals, etc.) using Raman imaging. Recent approaches have used surface-enhanced Raman spectroscopy (SERS) to investigate the structural changes of carotenoids and their interactions with other compounds and materials.

For a critical interpretation of the results obtained with Raman spectroscopy in accordance with the ethical standards of research, it is necessary to do the following: (a) select and test the best instrument parameters (for carotenoids, the laser intensity should be adjusted to minimize fluorescence, especially in samples masked by chlorophylls); (b) support the results via performing simultaneous measurements of the pure compounds (chemical standards) at different concentrations and of mixtures imitating the composition of carotenoids in a natural material; (c) perform standard analytical procedures (HPLC, HPTLC) on the same samples, especially in the case of the quantification of carotenoids serving as a correction factor; (d) carefully pre-process the Raman spectra (e.g., normalization, smoothing, and correction factors) on separate training and test spectra datasets; (e) select the appropriate chemometric model or combination thereof for the best interpretation of the Raman spectra, i.e., the identification and discrimination of different carotenoids present in a sample and/or their quantification. The future prospects of using RS in the study of carotenoids as model nutrients include the more precise discrimination of individual carotenoids in complex biological samples (e.g., foods, food products, plant extracts, human material for clinical research) thanks to the development of more sensitive Raman instruments, as well as the application of superior statistical models (e.g., machine learning, artificial intelligence). The use of better statistical techniques (e.g., machine learning, artificial neural networks) with subsequent external validation and model transfer approaches will increase the precision and repeatability of the chemometric studies performed. Advanced chemometric models are the “sine qua non” tool in the interpretation of Raman spectra, and therefore are absolutely necessary to the determination of individual and total carotenoids in complex matrices, as well as in the assessment of their distribution, transformation and quantification. SERS and 2D or 3D Raman imaging will be increasingly used in diagnostics as well as in clinical and pharmacological studies on the bioavailability and bioactivity of carotenoids.

Previously reported results confirm the optimistic expectations relating to the large-scale application of Raman spectroscopy in food quality control based on carotenoids-rich samples (e.g., freshness, ripeness, taste, color, shelf life). Finally, modern portable Raman instruments are preferable for the rapid in situ measurements of ripening, stress tolerance and varietal traits of carotenoids-rich plants, as well as for ex-tempore diagnostics associated with carotenoids as marker molecules. Although carotenoids are evolutionarily old pigments, and have been comprehensively studied for their multiple functions in plant life, as well as in human nutrition and health, there is still much work to be done in order to understand their complex behavior, structural modifications, and mechanisms of action in biological systems. We strongly believe in the development of Raman spectroscopy in conjunction with chemometrics due to its application potential in various disciplines. Future investigations should be oriented toward the application of new Raman techniques, such as SORS, CARS and SRS, to improve the quality of investigations and obtain more detailed and useful information.

## Data Availability

No new data were created or analyzed in this study. Data sharing is not applicable to this article.
